# Fast oscillatory activity in the anterior cingulate cortex: dopaminergic modulation and effect of perineuronal net loss

**DOI:** 10.3389/fncel.2014.00244

**Published:** 2014-08-20

**Authors:** Pascal Steullet, Jan-Harry Cabungcal, Michel Cuénod, Kim Q. Do

**Affiliations:** Department of Psychiatry, Center of Psychiatric Neuroscience, Centre Hospitalier Universitaire Vaudois and University of LausannePrilly-Lausanne, Switzerland

**Keywords:** anterior cingulate cortex, dopamine receptors, beta oscillations, perineuronal nets, parvalbumin interneurons, mouse

## Abstract

Dopamine release in the prefrontal cortex plays a critical role in cognitive function such as working memory, attention and planning. Dopamine exerts complex modulation on excitability of pyramidal neurons and interneurons, and regulates excitatory and inhibitory synaptic transmission. Because of the complexity of this modulation, it is difficult to fully comprehend the effect of dopamine on neuronal network activity. In this study, we investigated the effect of dopamine on local high-frequency oscillatory neuronal activity (in β band) in slices of the mouse anterior cingulate cortex (ACC). We found that dopamine enhanced the power of these oscillations induced by kainate and carbachol, but did not affect their peak frequency. Activation of D2R and in a lesser degree D1R increased the oscillation power, while activation of D4R had no effect. These high-frequency oscillations in the ACC relied on both phasic inhibitory and excitatory transmission and functional gap junctions. Thus, dopamine released in the ACC promotes high-frequency synchronized local cortical activity which is known to favor information transfer, fast selection and binding of distributed neuronal responses. Finally, the power of these oscillations was significantly enhanced after degradation of the perineuronal nets (PNNs) enwrapping most parvalbumin interneurons. This study provides new insights for a better understanding of the abnormal prefrontal gamma activity in schizophrenia (SZ) patients who display prefrontal anomalies of both the dopaminergic system and the PNNs.

## Introduction

Dopamine released in the prefrontal cortex plays a critical role in cognitive function such as working memory, attention and decision making (Seamans and Yang, 2004; Tritsch and Sabatini, 2012). D2R-binding PET studies suggest that dopamine is released in the prefrontal cortex during attention set-shifting (Ko et al., 2009), sustained attention and working memory task (Aalto et al., 2005), and during mild psychological stress (Lataster et al., 2011). Microinjection of dopamine receptor antagonists/agonists in rodent medial prefrontal cortex has revealed that D1R and D4R play a role in working memory (Zhang et al., 2004; Vijayraghavan et al., 2007), D1R and D2R regulate risk-based decision making (St Onge et al., 2011) and are necessary for attention set-shifting responses (Floresco et al., 2006), D4R and D1R differentially modulate encoding of salient and non-salient emotional information (Lauzon et al., 2009). Dopamine is therefore critical for proper processing of information within the prefrontal cortex during a number of cognitive tasks.

Through multiple types of receptors, dopamine exerts complex modulations on the excitability of pyramidal neurons and interneurons. It also regulates excitatory and inhibitory synaptic transmission at either pre- or postsynaptic loci. Moreover, the effect of dopamine varies among cell types, synapses, cortical layers and may depend on the level of neuronal activity (Seamans and Yang, 2004; Tritsch and Sabatini, 2012). Dopamine receptor activation in the prefrontal cortex can modify synaptic inputs to the cortical network (Gurden et al., 2000) and the local recurrent excitatory synapses (Onn et al., 2006). It can also selectively influence the strength of specific outputs to subcortical structures (Gee et al., 2012), increase the input-output responses in pyramidal neurons (Thurley et al., 2008), modulate persistent synaptic activity and enhance the signal-to-noise ratio (Kroener et al., 2009). Furthermore, the concentration of dopamine associated with its tonic or phasic release determines how this neuromodulator influences information processing via a predominant activation of either D1-or D2-type receptors. Seamans and Yang (2004) proposed a model in which multiple inputs in prefrontal cortex would access to the working memory buffers allowing multiple representations when D2R activation is predominant. By contrast, when D1R activation prevails, only strong inputs would produce active and stable network representations. However, due to the complexity of dopamine modulation, it is difficult to fully comprehend the effect of dopamine on prefrontal network activity. Because dopamine in the prefrontal cortex modulates the temporal dynamics of feed-forward inhibition (Tierney et al., 2008) and increases the excitability of fast-spiking interneurons (Gorelova et al., 2002; Tseng and O’Donnell, 2007), this neuromodulator could potentially modulate fast rhythmic synchronized neuronal activity, which occurs during many cognitive processes (Howard et al., 2003; Fan et al., 2007; Engell and McCarthy, 2010) favoring information transfer (Sohal et al., 2009), fast selection and binding of distributed neuronal responses (Fries et al., 2007). Local dopaminergic modulation of prefrontal γ oscillations has been proposed (Whittington et al., 2011; Furth et al., 2013), but experimental evidence for such a regulation is lacking.

In the present study, we investigated whether dopamine can modulate local fast rhythmic neuronal synchronization in slices of the anterior cingulate cortex (ACC), a region of the medial prefrontal cortex richly innervated by dopaminergic neurons (Descarries et al., 1987; Rivera et al., 2008) and affected in several psychiatric conditions (Fountoulakis et al., 2008; Fornito et al., 2009; Minzenberg et al., 2009; Chan et al., 2011; Frodl and Skokauskas, 2012). The ACC contributes to decision making and conflict monitoring (Botvinick, 2007), cost benefit analysis (Assadi et al., 2009) and empathy (Bernhardt and Singer, 2012). To date, the dopaminergic system in the ACC has been implicated in cost-based decision making (Schweimer and Hauber, 2006), tasks requiring sustained attention and working memory (Aalto et al., 2005) and attention set-shifting (Lumme et al., 2007; Ko et al., 2009). One of the functions of dopamine in the ACC might therefore be the modulation of high-frequency neuronal synchronization to control information processing during some of the above cognitive tasks. If so, an abnormal prefrontal dopaminergic system might contribute to the abnormal γ oscillations observed in patients with schizophrenia (SZ). Finally because there is a marked deficit of perineuronal nets (PNNs, specialized extracellular matrix enwrapping most parvalbumin-expressing fast-spiking interneurons which support high-frequency oscillations) in the prefrontal cortex of SZ patients (Mauney et al., 2013), we also examined the effect of PNN loss on fast oscillatory neuronal activity in the ACC.

## Methods

### Animals

Experiments were performed on adult (~3–5-month-old) C57Bl/6J mice and were approved by the Swiss Veterinary Office of the Canton de Vaud (Switzerland).

#### Surgery and chondroitinase ABC treatment

This experiment was designed to assess the effect of PNN removal on fast oscillatory activity in the ACC of adult mice. To do that, PNNs were enzymatically degraded in the ACC via an intracortical injection of chondroitinase ABC (ChABC, from *Proteus vulgaris*, Sigma-Aldrich, Switzerland). Mice were anesthetized with ketamine/xylasine (73/11.6 mg/kg, i.p.). Isoflurane was used to maintain the mice in a deep state of anesthesia throughout the surgical procedure. Bilateral craniotomy was performed (Bregma ~1.2, lateral ~0.25, depth ~1.25 mm) to inject 1 μl ChABC (50 U/ml; 0.1 μl/min) into one ACC and 1 μl vehicle solution PBS with 0.1% BSA) into the contralateral ACC. As analgesics, lidocaine (Wacker Chemie AG, Switzerland) was locally applied while buprenorphine (Temgesic, Essex Chemie AG, Switzerland) was injected (0.1 mg/kg, s.c.) during surgery. Mice were sacrificed 3 days post-injection for electrophysiological and subsequent morphological assessment.

#### Electrophysiology

Anesthetized mice were perfused with oxygenated sucrose-containing artificial cerebrospinal fluid (ACF) (in mM: 252 sucrose, 3 KCl, 2 MgSO_4_, 1.2 CaCl_2_, 1.2 NaH_2_PO_4_, 24 NaHCO_3_, 10 glucose; pH 7.4) for 10 min prior to decapitation. Paracoronal slices (400 μm thick, Bregma ~1.4–0.6) containing the ACC were prepared with a vibroslicer in cold oxygenated sucrose-containing ACF, transferred into a “Haas” type interface chamber (kindly provided by MA Whittington) and superfused with oxygenated normal ACF (in mM: 126 NaCl, 3 KCl, 1 MgCl_2_, 1.2 CaCl_2_, 1.2 NaH_2_PO_4_, 24 NaHCO_3_, 10 glucose; pH 7.4). ACF temperature was slowly raised from room temperature to ~32°C. Electrophysiological recordings were performed at least 90 min after slicing. Field potentials were recorded with ACF-filled glass electrodes (~1 MΩ). Signals were band pass-filtered at 0.3–3000 Hz and digitized at 5 kHz. Oscillatory neuronal activity was generated with a mixture of kainate (0.8 μM) and carbachol, (50 μM) in 5 mM KCl-containing ACF. The recording electrode was positioned in the superficial part of layer 5 where the oscillatory activity was most powerful. Typically, a 10–15-min superfusion with kainate + carbachol was necessary to observe stable high-frequency oscillatory activity in the β band. This fast oscillatory neuronal activity vanished after removal of carbachol + kainate, but could be induced again to similar levels with these pharmacological agonists. Power spectrum analyses were performed on 60-s recordings using the Welch method (IgorPro6 WaveMetrics, Portland, OR, USA). The power density of fast oscillatory activity was calculated within the β band (13–28 Hz). Dopaminergic modulation of this rhythmic neuronal activity was assessed by comparing within the same ACC slice the power and peak frequency of the oscillations induced by carbachol + kainate with those generated by carbachol + kainate in the presence of dopaminergic agonist/antagonist. The concentration of the agonist of a given dopamine receptor type was chosen on the basis of its published constant Ki values for each type of dopaminergic receptors, so the concentration used (0.4–1 μM) would activate most of its specific receptors without exciting a large proportion of other dopaminergic receptor types.

We also investigated the contribution of gap junctions, GABAergic and glutamatergic receptors on the generation and maintenance of these local fast neuronal oscillations induced by carbachol + kainate and modulated by dopaminergic agonists. To do so, pharmacological blockers (carbenoxolone, picrotoxin, SYM2206, AP-5) were added to the superfusion after robust and stable oscillations were induced with quinpirole + kainate + carbachol. The power and peak frequency of the oscillations before and after adjunction of these pharmacological blockers were compared.

To study the effect of PNN loss on oscillations, we recorded and analyzed oscillations induced by co-application of quinpirole, kainate and carbachol in the ACC that were previously intracortically injected with ChABC and in the corresponding contralateral vehicle-injected ACC (sham). Recordings were performed bilaterally in 3–4 slices at and contiguous to the injection sites. After the recordings, each slice (400 μm thick) was then fixed in 4% paraformaldehyde and re-cut into 40 μm frozen sections for immunolabeling. Two non-contiguous sections of 400 μm slice were processed for immunofluorescence to check for qualitative density of PNN which was labeled with the lectin Wisteria Floribunda Agglutinin (WFA). Physiological data from slices displaying no or reduced WFA labeling in the ChABC-injected ACC compared to the contralateral sham ACC were analyzed together. Data from slices displaying no difference of WFA labeling in the ChABC-injected and vehicle-injected sides (slices posterior or anterior to the injection site) were also analyzed separately and used as an additional control dataset.

#### Immunofluorescence

Brain sections containing the ACC were first incubated with PBS + Triton 0.3% + sodium azide (1 g/L) containing 2% normal horse serum, then placed for 48 h in a solution with a rabbit polyclonal anti-parvalbumin (1:2500; Swant, Switzerland) primary antibody together with the biotin-conjugated lectin Wisteria Floribunda Agglutinin (WFA, 1:2000; Sigma, Switzerland). Sections were then washed, incubated with fluorescent secondary antibody conjugates (goat anti-rabbit IgG (1:300; CY3; Chemicon International, USA) and streptavidin CY2 conjugate (1:300; Millipore Corporation, USA)), and counterstained with 100 ng/ml DAPI (4′-6-diamidino-2-phenylindole; Vector Laboratories Incorporation, California, USA).

#### Statistical analysis

Effects of pharmacological agents on oscillations (power and peak frequency) were assessed using paired Student *t*-test (for each recorded ACC slice, comparison between oscillations in absence and presence of the pharmacological agent). When data was not normal based on a Kolmogorov-Smirnov test, Wilcoxon-signed rank test was used. Bonferroni correction was applied when more than two conditions were tested in a same ACC slice and more than one pair comparisons were performed. The effect of PNN degradation by ChABC on oscillations was analyzed using paired Student *t*-test (comparison of oscillations on the ChABC-side and on the contralateral sham side of a same slice).

#### Chemicals

Carbachol (carbamylcholine chloride), kainate (kainic acid monohydrate), quinpirole, (+)- sulpiride, (+)-SKF-38393, PD168,077, carbenoxolone disodium salt, picrotoxin were purchased from Sigma (Sigma-Aldrich, Buchs, Switzerland); SYM2206 and L745.870 were from Tocris Bioscience (Bristol, UK); AP-5 (DL-2-amino-5-phosphopentanoic acid) was from Alexis Biochemicals (San Diego, CA); R(+)-SCH23390 HCl was from Research Biochemical International (Sigma-Aldrich, Buchs, Switzerland).

## Results

### Activation of dopamine receptors (D2R and D1R) increases local fast oscillatory activity in the ACC

**Figure 1:**
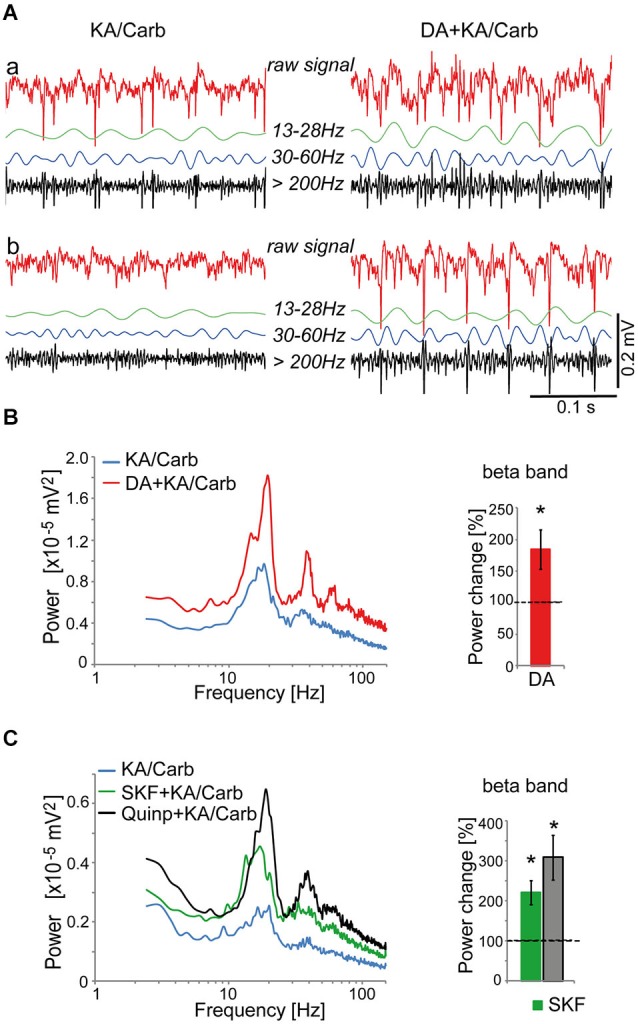
**Dopaminergic modulation of high-frequency oscillations in the anterior cingulate cortex (ACC). (A)** Illustration of recordings from two ACC slices (a and b) superfused with kainate + carbachol (KA/Carb) in absence (left) and presence (right) of 50 μM dopamine (DA). For each recording, the raw unfiltered signal (red), the filtered signals in the β band (13–28 Hz, green), γ band (30–60 Hz, blue) and high-frequency band (>200 Hz, black) are displayed. **(B)** DA significantly enhances the power of high-frequency oscillations (in the β band) induced by KA/Carb (two-tailed, *P* = 0.008, *n* = 8). *Left*: power spectra of recordings (mean of pooled data). *Right*: DA-induced change in the power of the oscillations (100% corresponds to the power of oscillations elicited by KA/Carb in absence of DA). * *P* < 0.05, significantly different from the KA/Carb condition. **(C)** The D2R agonist (quinpirole, Quinp, 1 μM) and the D1R agonist (SKF-38393, SKF, 0.8 μM) significantly enhance the power of the oscillations induced by KA/Carb (two-tailed, for Quinp: *P* = 0.003; for SKF: *P* = 0.004, *n* = 11). *Left*: power spectra of recordings (mean of pooled data). *Right*: Change in the power of the oscillations induced by Quinp and SKF, respectively (100% corresponds to the power of oscillations elicited by KA/Carb in absence of DA agonists). * *P* < 0.05, significantly different from the KA/Carb condition after Bonferroni correction. Bars, sem.

To investigate the potent dopaminergic modulation of fast rhythmic neuronal synchronization, we first searched for a pharmacological method to induce persistent fast neuronal oscillations in slices of the ACC, more precisely in the cg1 area (Franklin and Paxinos, 2008). This region is homologous to the human Brodmann area 24b (Vogt and Paxinos, 2014). A mixture of kainate (0.8 μM) and carbachol (50 μM) generated small and stable neuronal oscillations in the β frequency band, reflecting fast rhythmic neuronal synchronization. We evaluated the effect of dopamine on these oscillations by comparing, within the same slices, the oscillatory activity induced by kainate + carbachol with and without dopamine. Dopamine (50 μM) significantly enhanced the power of these oscillations (Figures 1A,B) but did not affect their peak frequencies (mean ± SD, 17.3 ± 1.3 Hz vs. 17.2 ± 1.9 Hz with dopamine, *n* = 18).

We then screened for the dopamine receptor type(s) responsible for the modulation of these oscillations. Both the D2R-type agonist, quinpirole (0.5–1 μM), and the D1R agonist, SKF-38393 (0.8 μM), significantly enhanced the power of these oscillations (Figure 1C) without affecting their peak frequencies (17.3 ± 1.9 Hz vs. 16.5 ± 2.4 Hz with SKF-38393, *n* = 21; 18.1 ± 2.1 Hz vs. 18.6 ± 2.2 Hz with quinpirole, *n* = 30). However, the power enhancement induced by SKF-38393 tended to be weaker than that mediated by quinpirole. Thus, in 8 out of 11 ACC slices where both agonists were tested, the power of oscillations was stronger in the presence of quinpirole than with SKF-38393.

**Figure 2:**
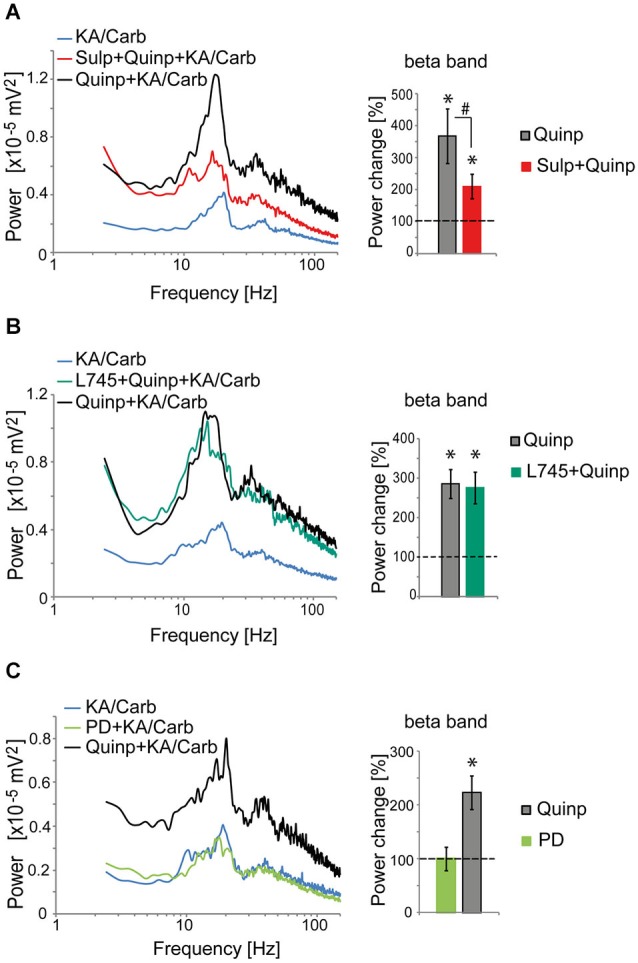
**Activation of D2R, but not D4R, enhances the power of high-frequency oscillations in the ACC**. **(A)** Activation of D2R enhances the power of the oscillations induced by kainate + carbachol (KA/Carb) (one-tailed, *P* = 0.001, *n* = 12). The D2R antagonist (sulpiride, Sulp, 10 μM) significantly reduces the power increase induced by quinpirole (Quinp, 1 μM) (one-tailed, *P* = 0.001). However, Sulp does not fully prevent the Quinp-induced increase in power (Quinp + Sulp vs. no D2R modulator condition: one-tailed, *P* = 0.007).* Left*: power spectra of recordings (mean of pooled data). *Right*: Change in the power of the oscillations induced by Quinp and by Quinp + Sulp, respectively (100% corresponds to the power of oscillations elicited by KA/Carb in absence of dopaminergic modulators). * *P* < 0.05, significantly different from the KA/Carb condition after Bonferroni correction; ^#^ *P* < 0.05, significantly different between the two conditions after Bonferroni correction. **(B)** The D4R antagonist (L745.870, L745, 10 μM) does not decrease the Quinp-induced enhancement of the power of oscillations elicited by KA/Carb (Quinp vs. Quinp + L754: *P* > 0.05, *n* = 15). Quinp as well as Quinp + L745 increase significantly the power of oscillations elicited by KA/Carb (for both, one-tailed, *P* < 0.0005).* Left*: power spectra of recordings (mean of pooled data). *Right*: Change in the power of the oscillations induced by Quinp and by Quinp + L745, respectively (100% corresponds to the power of oscillations elicited by KA/Carb in absence of dopaminergic modulators). * *P* < 0.05, significantly different from the KA/Carb condition after Bonferroni correction. **(C)** Activation of D4R does not enhance the power of the oscillations that are induced by KA/Carb. While Quinp significantly increases the power (one-tailed, *P* = 0.009, *n* = 7), the D4R agonist (PD168.077, PD, 0.4 μM) does not (one-tailed, *P* > 0.05). *Left*: power spectra of recordings (mean of pooled data). *Right*: Change in the power of the oscillations induced by Quinp and PD, respectively (100% corresponds to the power of oscillations elicited by KA/Carb in absence of DA agonists). * *P* < 0.05, significantly different from the KA/Carb condition after Bonferroni correction. Bars, sem.

**Figure 3:**
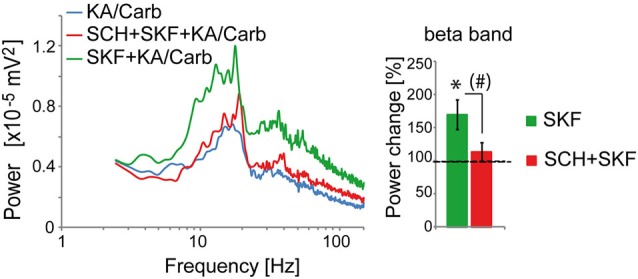
**Activation of D1R enhances the power of high-frequency oscillations in the ACC**. The enhancement of the power of the oscillations that is induced by the D1R agonist (SKF-38393, SKF, 0.8 μM) (one-tailed, *P* = 0.0085, *n* = 10) is prevented by the D1R antagonist (SCH23390, SCH, 5 μM). SCH reduces the power increase induced by SKF (one-tailed, *P* = 0.02) and the power is not significantly higher in the SKF + SCH compared to the no D1R modulator condition (one-tailed, *P* > 0.05). *Left*: power spectra of recordings (mean of pooled data). *Right*: Change in the power of the oscillations induced by SKF and by SKF + SCH, respectively (100% corresponds to the power of oscillations elicited by KA/Carb in absence of dopaminergic modulators). * *P* < 0.05, significantly different from the KA/Carb condition after Bonferroni correction; ^#^ *P* < 0.05, but no more significantly different between the two conditions after Bonferroni correction. Bars, sem.

The D2R specific antagonist, sulpiride (10 μM), prevented most of the quinpirole-induced enhancement of the oscillations (Figure 2A). In contrast, the D4R antagonist, L745.870 (10 μM), did not affect the power of the oscillations induced by quinpirole + kainate + carbachol (Figure 2B), indicating that D2R but not D4R activation causes enhancement of these high-frequency oscillations. The inefficiency of D4R was further confirmed as the specific D4R agonist, PD168.077 (0.4 μM), did not alter neither the power (Figure 2C) nor the frequency of the oscillations (18.7 ± 1.9 Hz vs. 18.8 ± 2.2 with PD168.077, *n* = 7) induced by kainate + carbachol. The selective D1R antagonist, SCH23390 (5 μM), prevented the power increase induced by SKF-38393 (Figure 3), confirming that D1R activation also enhances these oscillations in the ACC. Taken together, the data demonstrated a role of D2R and D1R receptors in the modulation of persistent fast oscillatory activity in the local cortical circuitry of the ACC.

### Fast oscillatory activity in the ACC requires GABA_A_ receptors, AMPA receptors and gap junctions

**Figure 4:**
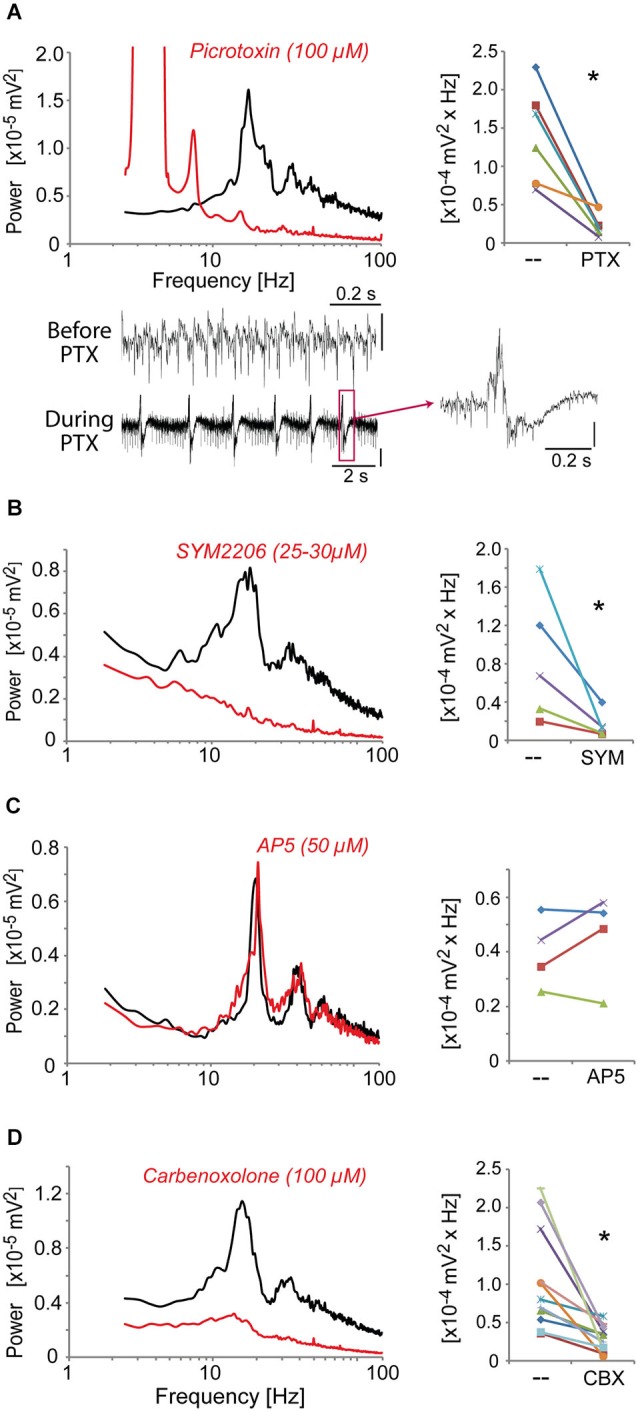
**The fast oscillatory activity that is elicited by a mixture of quinpirole, kainate and carbachol (Quinp/KA/Carb) requires activation of GABA_A_ receptors, AMPA receptors, and functional gap junctions. (A)** Inhibition of GABA_A_ receptors (with picrotoxin, PTX, 100 μM, for 30 min) abolishes the high-frequency oscillations (one-tailed, *P* = 0.0025, *n* = 6). Traces show recordings before and during PTX application. Note the regular epilepticform activity following PTX superfusion. The right trace shows one of these epileptic-like events. Vertical scales: 0.1 mV. **(B)** Inhibition of AMPA receptors (with SYM2206, SYM, 25–30 μM, for 40 min) abolishes the oscillations (one-tailed, *P* = 0.023, *n* = 5). **(C)** Inhibition of NMDA receptors (with AP5, 50 μM, for 40 min) does not affect the oscillations (one-tailed, *P* > 0.05, *n* = 4). **(D)** Functional impairment of gap junctions (with carbenoxolone, CBX, 100 μM, for 60 min) abolishes the oscillations (one-tailed, *P* = 0.007, *n* = 11). *Left* graphs: power spectra of recordings (mean of pooled data) during superfusion with Quinp/KA/Carb in the absence (black) or presence (red) of the drugs. Note for the right graphs: the power values in the β band recorded in the same ACC before and after application of the drugs are connected by a line. * *P* < 0.05.

We then identified the neurotransmitter systems and receptors required to generate these high-frequency oscillations induced in the ACC by kainate + carbachol and modulated by dopaminergic receptor agonists. Blocking GABA_A_ receptors with picrotoxin (50 μM) fully disrupted the oscillations generated by quinpirole, kainate and carbachol (Figure 4A) and instead led to epilepticform activity (present in 6 out of 8 slices) (Figure 4A). The selective AMPA receptor antagonist, SYM2206 (25–30 μM), also abolished these oscillations indicating the requirement of AMPA receptor activation (Figure 4B). In contrast, blocking NMDA receptors with AP-5 (50 μM) did not alter these oscillations (Figure 4C). Carbenoxolone (100 μM), a compound known to impair the function of gap junctions, also disrupted the oscillations (Figure 4D). Together, these data indicated that this fast oscillatory activity in the ACC is generated within a local network of excitatory and inhibitory neurons requiring functional gap junctions, phasic AMPA receptor-dependent excitation, and GABA_A_ receptor-dependent inhibition.

### Absence of intact perineuronal nets enhances the power of high-frequency oscillations

**Figure 5:**
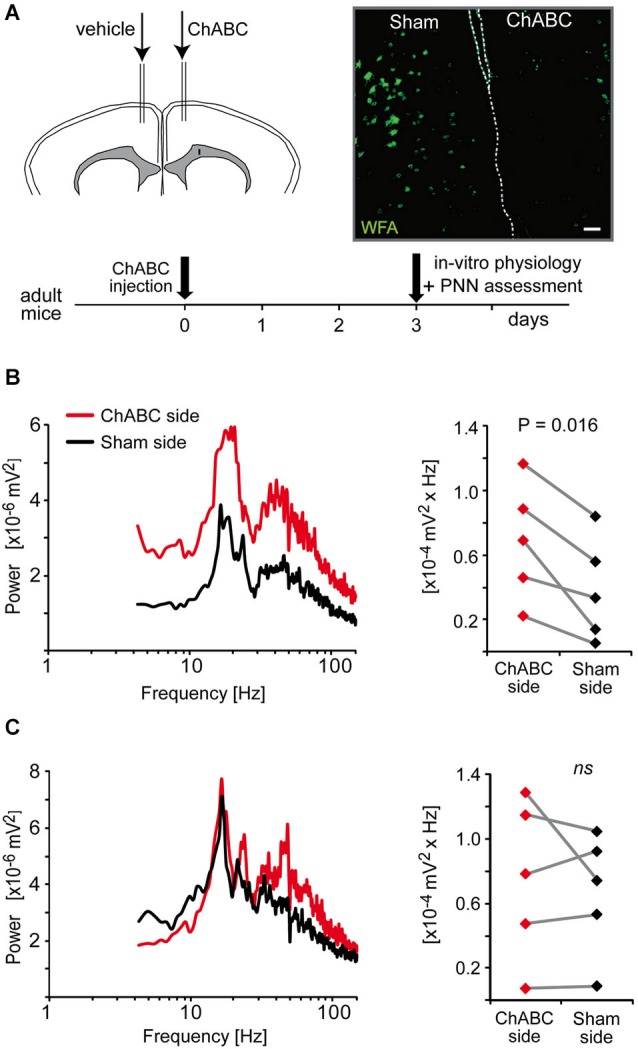
**Enzymatic removal of the perineuronal nets (PNNs) by local injection of chondroitinase ABC (ChABC) increases the power of high-frequency oscillations induced by a mixture of quinpirole, kainate and carbachol (Quinp/KA/Carb). (A)** Schema of the experimental protocol of ChABC and control vehicle injections followed by electrophysiological and histological assessment. Micrograph illustrates the integrity of PNNs (as assessed with WFA labeling) in the vehicle-injected side (sham ACC, *left*) and in the ChABC-injected side (*right*). Dashed lines indicate separation between hemispheres. Scale: 80 μm. **(B)** In coronal slices that show a degradation of PNNs in the ChABC-injected ACC (as assessed by the WFA labeling), the power of the oscillations is significantly larger in the ChABC-injected ACC compared to the contralateral control ACC (sham-injected). **(C)** In coronal slices that are anterior or posterior to the ChABC injection site (the PNNs remain intact as assessed by WFA labeling), the power of the oscillations in the ACC of the two hemispheres is not significantly different from each other. *Left* graphs: power spectra of recordings from the ChABC-injected ACC (red) and from the contralateral sham ACC (black) (mean of pooled data). Note for the right graphs: the power values in the β band recorded in the ChABC-injected side and the control sham side from the same brain slice are connected by a line.

Results from a previous study suggest that the fast rhythmic synchronized activity, which can be induced by quinpirole, kainate and carbachol in the ACC, is dependent on parvalbumin interneurons (Cabungcal et al., 2013). Indeed, a deficit in these fast oscillations was associated with a reduced number of parvalbumin-immunoreactive neurons in fully adult *Gclm* KO mice (Cabungcal et al., 2013), which have a limited capacity to produce the antioxidant glutathione (Steullet et al., 2010). Most matured parvalbumin interneurons are enwrapped by a specialized extracellular matrix, the PNNs, which consists of chondroitin sulfate proteoglycans (e.g., versican, aggrecan, neurocan, brevican), hyaluronan, tenascin and link proteins (Kwok et al., 2011). These PNNs promote synaptic and network stability, are involved in the maturation and phenotypic maintenance of parvalbumin interneurons and protect them against oxidative stress (Sugiyama et al., 2009; Kwok et al., 2011; Cabungcal et al., 2013). On this basis, we examined whether PNNs play a role in the maintenance of the oscillatory activity induced by quinpirole, kainate and carbachol. The PNNs were degraded unilaterally in one ACC of adult mice by a local injection of chondroitinase ABC (ChABC), an enzyme that breaks down chondroitin sulfates and hyalorunan. This treatment led to absence of labeling by WFA (*Wisteria Floribunda Agglutinin)*, a lectin that preferentially binds to the PNNs around parvalbumin interneurons (Figure 5A). About 3-days post-injection, mice were sacrificed and electrophysiological recordings were performed on slices containing the ChABC-injected ACC and its contralateral control Sham ACC. The mixture of quinpirole, kainate and carbachol induced high-frequency oscillations in both ChABC-injected and Sham ACC. The power of the oscillations was however significantly higher in the ChABC-injected ACC (which display no WFA labeling as verified *post-hoc* by immunofluorescence) than in their respective contralateral sham ACC (which show normal WFA labeling) (Figure 5B). By contrast, in slices anterior or posterior to the ChABC injection site where the PNNs remained intact, the power of oscillations was not significantly different between the ACC of both hemispheres (Figure 5C). This indicated that the absence of intact PNNs around parvalbumin interneurons does not disrupt the fast rhythmic activity in the ACC, but rather enhances the power of the oscillations associated with this network activity. The effect of PNN degradation on the power of the oscillations further supports a role for parvalbumin interneurons in this fast oscillatory neuronal activity in the ACC.

## Discussion

In the present study, we show that the power of fast oscillatory activity induced by kainate + carbachol is increased by D2R and D1R activation in ACC slices of mice. These oscillations require functional AMPA receptors, GABA_A_ receptors and gap junctions, and are enhanced in absence of intact PNNs.

The enhancement of local fast oscillatory neuronal activity by dopamine suggests that this neuromodulator improves information transfer (Sohal et al., 2009) and fast selection and binding of distributed neuronal responses (Fries et al., 2007) within the ACC. Interestingly, activation of either D1R or D2R gives rise to a similar modulation of these oscillations. This contrasts with the usual opposite effects of these two types of receptors on pyramidal neuron excitability, GABAergic and glutamatergic synaptic transmission (Tritsch and Sabatini, 2012) and even coupling between neurons (Onn and Grace, 1994). This suggests that dopamine-induced enhancement of high-frequency synchronization is a primordial function in the ACC and may be essentially driven by fast-spiking interneurons whose excitability is increased by the activation of both D1R and D2R receptors (Gorelova et al., 2002; Tseng and O’Donnell, 2007; Towers and Hestrin, 2008). Other data further support a central role of fast-spiking interneurons in the generation of these fast oscillations. Thus, we found a negative correlation between the power of these oscillations and the number of parvalbumin-immunoreactive interneurons in the ACC of *Gclm* KO mice, an animal model of redox dysregulation and vulnerability for oxidative stress in SZ (Cabungcal et al., 2013). The number of parvalbumin interneurons and the power of these oscillations were not different in young adult *Gclm* KO and WT mice, while in older individuals a decrease in number of parvalbumin-immunoreactive interneurons in *Gclm* KO mice was associated with impaired oscillations. Moreover, our present work shows that enzymatic degradation of the PNNs that enwrap most parvalbumin interneurons also affects these oscillations. Since PNNs are involved in synaptic and network stability (Sugiyama et al., 2009; Kwok et al., 2011), we expected that PNN degradation might disrupt fast neuronal synchronization. Instead, we observed an enhanced power of these high-frequency oscillations. These results are however in line with the power increase in β/γ oscillations observed in mice deficient in tenascin-R, a component and stabilizer of PNNs (Gurevicius et al., 2004). Because PNNs act as a cation buffer via their polyanionic nature, they may slow down or reduce cation exchange through membrane ion channels and therefore limit the interneuron excitability. Indeed, PNN degradation with ChABC lowered the excitability threshold of fast-spiking interneurons (Dityatev et al., 2007), which could result in stronger β/γ oscillations.

While the local fast neuronal synchronization in the ACC critically depends on GABAergic neurons, as blocking GABA_A_ receptors fully disturbs high-frequency oscillations, excitatory pyramidal neurons are also necessary. Indeed, blocking AMPA receptors abolishes these oscillations. Thus, the fast oscillations in the ACC that are modulated by dopamine are generated by a local network of excitatory and inhibitory neurons (including parvalbumin interneurons) and require gap junctions and both phasic AMPA receptor-dependent excitation and GABA_A_ receptor-dependent inhibition. The type of neuronal network engaged in the persistent high-frequency oscillations in the ACC resembles those found in the auditory cortex (Roopun et al., 2010) and in the superficial layers of the somatosensory cortex (Roopun et al., 2006), but differs from that of the motor cortex (Yamawaki et al., 2008) or the deep layer of the somatosensory cortex (Roopun et al., 2006). Indeed, the pharmacologically-induced persistent fast oscillations in the motor cortex did not depend on AMPA receptors and the β oscillations in the deep layer of the somatosensory cortex did not require AMPA and GABA_A_ receptors.

Dopamine modulation of fast rhythmic neuronal synchronization has been suggested by several authors (Whittington et al., 2011; Furth et al., 2013) and simulated from artificial neuronal networks (Kuznetsova and Deth, 2008; Kömek et al., 2012). Dopamine modulation of γ oscillations has been described in the hippocampus (Weiss et al., 2003; Wójtowicz et al., 2009; Andersson et al., 2012). However, the dopaminergic modulation of high-frequency local neuronal synchronization in the neocortex has not been much investigated. DRD4 and DAT1 polymorphisms modulate auditory-evoked γ responses in humans, suggesting a dopaminergic modulation of cortical networks (Demiralp et al., 2007). Recently, Kocsis et al. (2013) have shown that administration of a D4R agonist increases γ oscillations in hippocampus and several cortical regions (including prefrontal cortex) in awake rats. However, D4R activation did not enhance the high-frequency oscillations induced by kainate + carbachol within a local neuronal network of the ACC. The network of neurons engaged in a high-frequency synchronized activity in slices via pharmacological activation of cholinergic and glutamatergic receptors might be different from the one engaged *in-vivo*. The cortical activity *in-vivo* is driven and modulated by the afferents of many brain regions. Therefore, we cannot exclude that the increase of prefrontal γ oscillations following injection of a D4R agonist may be primarily due to the action of D4R in other regions, and not to a direct modulation of the local cortical network. Moreover, the direction and the nature (type of receptors involved) of the local dopaminergic modulation of high-frequency oscillations vary across brain regions and even across subregions of the medial prefrontal cortex. Thus, dopamine increases and decreases high-frequency oscillatory activity in the prelimbic and ventral infralimbic cortex, respectively (Glykos et al., 2012). On the other hand, the power of γ oscillations in the hippocampus is enhanced by D4R activation, while D1/5R activation has an opposite effect (Weiss et al., 2003; Wójtowicz et al., 2009; Andersson et al., 2012). The dopamine-induced changes of synaptic and spike activity in ACC pyramidal neurons and interneurons that lead to an increase of fast oscillations remain however undetermined. Since D1R and D2R activation promote the firing of prefrontal fast-spiking interneurons (Gorelova et al., 2002; Tseng and O’Donnell, 2007; Towers and Hestrin, 2008), dopamine might recruit an increasing number of these interneurons and enhance their synchronization as observed in the hippocampus (Andersson et al., 2012). A more in depth study to elucidate how dopamine in the ACC modulates synaptic input integration and the dynamic of spike generation in pyramidal neurons and interneurons during fast oscillatory activity is needed to better understand the dopamine role in information processing within the local ACC neuronal circuit.

The ACC is part of the attention executive network. This prefrontal region links sensory information with rules or expectations to generate motor responses. It is implicated in learning and predicting the likely outcome of actions through evaluation of the probable and actual outcomes of one’s action (Alexander and Brown, 2011) or actions from other individuals (Rushworth et al., 2007). The dopaminergic system in the ACC plays a role in cost-based decision making (Schweimer and Hauber, 2006), cognitive tasks such as attention set-shifting tasks (Lumme et al., 2007; Ko et al., 2009) and sustained attention and working memory tasks (Aalto et al., 2005). Therefore, it is quite plausible that, in the ACC, dopamine improves the flow of information processing that is associated with local fast neuronal synchrony during tasks requiring attention, working memory and decisions (Howard et al., 2003; Engell and McCarthy, 2010).

In psychiatric diseases such as SZ, the ACC is affected and its activation often abnormal (Dolan et al., 1995; Minzenberg et al., 2009; Kyriakopoulos et al., 2012). Specifically, attention executive function, decision making and cost benefit analysis that all require the ACC are impaired in patients. Interestingly, the dopaminergic system in the ACC is also altered, with increased D1R binding (Abi-Dargham et al., 2012) and decrease D2R binding (Suhara et al., 2002) in drug-naïve SZ compared to healthy subjects. A line of indirect evidence also suggests that SZ is characterized by a hyperfunction and hypofunction of the dopaminergic system in the striatum and the prefrontal cortex, respectively (Simpson et al., 2010). Noteworthy, the failure of a cognitive task to induce an increase in cerebral blood flow in the ACC of SZ patients can be significantly recovered by the dopaminergic agonist, apomorphine (Dolan et al., 1995; Fletcher et al., 1996). Because haemodynamic signals correlate with the power of γ oscillations (Niessing et al., 2005; Mulert et al., 2010; Scheeringa et al., 2011), it is therefore possible that abnormal dopaminergic transmission in the ACC and other prefrontal regions contributes to abnormal modulation of induced γ oscillations, as observed in patients. Basar-Eroglu et al. (2007) found that the power of γ oscillations in patients fails to be enhanced during a high-demanding working memory task. Likewise, the induced γ oscillations during a high cognitive control tasks were impaired in frontal areas of patients (Cho et al., 2006). By contrast, the power of baseline frontal γ oscillations in patients is often higher compared to healthy controls (Basar-Eroglu et al., 2007; Barr et al., 2010; Bandyopadhyaya et al., 2011; Gandal et al., 2012). Intuitively, these strong spontaneous prefrontal γ oscillations in patients reveal an apparent contradiction with the anomalies of the network associated with the fast-spiking parvalbumin-expressing interneurons (Lewis et al., 2011). Some data suggest however that a default in NMDAR function in fast-spiking interneurons could contribute to enhanced spontaneous γ oscillations in patients. Indeed, a study reports a drastic decrease in number of GAD67-positive neurons expressing the NMDA receptor subunit NR2A in prefrontal cortex of patients (Woo et al., 2008) and a lack of NMDAR neurotransmission in parvalbumin cells via a specific deletion of NR1 on these cells leads to enhanced baseline cortical γ rhythms (Carlén et al., 2012). On the other hand, it is also plausible that reduced or abnormal PNNs in prefrontal cortex of patients (Mauney et al., 2013) could contribute to the enhanced baseline frontal γ rhythms that are sometimes observed in patients.

To conclude, we have demonstrated a robust dopaminergic modulation of local high-frequency oscillations in the ACC and an enhanced power of these oscillations in absence of intact PNNs. Our data provide new insights on the modulation of high-frequency neuronal synchronization in the prefrontal cortex and may bring a novel perspective for understanding the abnormal γ oscillations in the frontal cortex of SZ patients.

## Author contributions

Pascal Steullet, Jan-Harry Cabungcal, and Kim Q. Do designed research; Pascal Steullet performed and analyzed electrophysiological experiments; Jan-Harry Cabungcal performed surgery and immunofluorescence; Pascal Steullet wrote the paper; all authors critically revised the manuscript.

## Conflict of interest statement

The authors declare that the research was conducted in the absence of any commercial or financial relationships that could be construed as a potential conflict of interest.
